# It’s Raining, It’s Pouring, the Old Man Is Snoring: Content Analysis of Age Stereotypes in Nursery Rhymes

**DOI:** 10.2196/70841

**Published:** 2026-02-17

**Authors:** Reuben Ng, Nicole Indran

**Affiliations:** 1 Lee Kuan Yew School of Public Policy, National University of Singapore, 469C Bukit Timah Road, Singapore, 259772, Singapore, 65 66015229

**Keywords:** portrayals of old age, ageism, reframing aging, text as data, psychomics, age stereotypes

## Abstract

**Background:**

Ageist beliefs tend to take root in one’s formative years and persist into adulthood, making it crucial to unpack the ways in which children are socialized to view old age. This study is the first to analyze portrayals of old age in nursery rhymes. Related literature has concentrated largely on depictions of older adults in books or movies targeted at children. As a staple of early childhood education, nursery rhymes merit examination as a vehicle through which age stereotypes are disseminated and reinforced.

**Objective:**

Our content analysis of nursery rhymes is grounded in 3 research questions: to what extent is old age represented in nursery rhymes? What are the prevailing stereotypes associated with old age in nursery rhymes? Are these stereotypes primarily positive or negative?

**Methods:**

To build a comprehensive dataset, we gathered material from the following websites, each of which houses an extensive collection of rhymes, including BBC Nursery Rhymes and Songs, the Nursery Rhyme Collections, All Nursery Rhymes, and NurseryRhymes.org. A web ingestion tool was used to compile the data. In total, 735 unique nursery rhymes were retrieved. To identify rhymes related to old age, we conducted a search using various terms related to old age (eg, old, aged, grandfather, and grandma), which yielded 85 nursery rhymes. After applying a rigorous set of exclusion criteria, 29 rhymes remained. Both deductive and inductive modes of reasoning guided our content analysis.

**Results:**

Old age was a central theme in 4% (N=29) of the 735 nursery rhymes. Of the 29 rhymes analyzed, more than half contained negative age stereotypes (17/29, 59%), and a third (10/29, 34%) contained positive ones. A small proportion portrayed old age in a neutral manner (2/29, 7%). Examples of negative stereotypes include being physically debilitated, cognitively impaired, helpless, unhygienic, and incompetent. Examples of positive stereotypes include being wise, affectionate, and jovial. Neutral portrayals framed aging as a natural part of life.

**Conclusions:**

In the context of an aging population, it is paramount that people embrace a less pessimistic outlook on aging. Although nursery rhymes may seem like mere tales not to be taken seriously, they are powerful cultural artifacts capable of molding thought processes. Our study highlights the need to give children access to more accurate and nuanced stories about older adults. By doing so, society can move beyond passively assimilating negative views of aging to actively fostering healthier ones, thus building a future where all are valued regardless of age.

## Introduction

### Background

Ageism refers to stereotyping, discrimination, and prejudice on the grounds of age [1,2]. Globally, one in two individuals aged 16 years and older exhibits ageist attitudes toward older adults [1]. Such ageist beliefs tend to take root in one’s formative years and persist into adulthood [3,4], making it crucial to unpack the ways in which children are socialized to view old age. In this study, we analyze depictions of old age in nursery rhymes.

Nursery rhymes are traditional poems, lullabies, or songs typically read or sung to children with the intention of instilling in them positive behaviors [5]. Most well-known nursery rhymes were penned decades or centuries ago but continue to be a staple of early childhood education [6]. On top of being entertaining, nursery rhymes support multiple aspects of a child’s development [6], which could explain their enduring appeal. Today, nursery rhymes are enjoyed not only through books but also through television shows and videos, where they have amassed billions of views.

### Theoretical Background

Central to the theory of cultivation is the notion that prolonged exposure to the media leads the individual to imbibe the views and values depicted [7,8]. Though originally formulated to elucidate the impact that television can have on viewers, the theory has since evolved to encompass different forms of media, including movies and books [9]. An important aspect of the theory of cultivation is the idea of mainstreaming, which posits that constant exposure to the media can result in a homogenization of perspectives. More specifically, through the process of mainstreaming, audiences who would otherwise hold very different views of the world come to assume a common view of reality [10]. There is evidence that the more people watch television programs that propagate negative messages about older adults, the more they perceive older adults unfavorably [11].

The communicative ecology model of successful aging postulates that perceptions of aging are derived from the routine ways in which aging is discursively constructed, whether through direct or mediated communication [12]. A key plank of this model is that uncertainty about aging influences one’s sense of self-efficacy in navigating the aging process. As a form of early socialization, nursery rhymes contribute to this broader communicative ecology, molding perceptions of aging from a young age. When negative messages about aging are conveyed through these rhymes, they may induce feelings of uncertainty about later life. Meanwhile, positive depictions of aging can bolster one’s sense of self-efficacy and promote more empowering attitudes toward later life [13].

There is evidence of a general prejudice against older adults among children [14-16]. Negative stereotypes of older persons have been observed in children as young as 3 years old [14,15]. By the time they reach elementary school, they may even verbalize these stereotypes [17]. The theory of stereotype embodiment submits that age stereotypes are internalized throughout one’s life span and eventually become self-stereotypes that affect one’s health. It is well documented that the assimilation of negative age stereotypes into one’s self-concept correlates with poorer health outcomes such as a higher risk of depression and a shorter lifespan [3,18-21]. Meanwhile, positive age stereotypes are associated with improved functional health and overall well-being [3,18,19]. The types of stereotypes espoused early in one’s life matter too. Research shows that younger individuals who hold negative age stereotypes are more likely to experience a cardiovascular event in later life than those with positive stereotypes [22]. In addition to being a threat to public health, ageism also constitutes a major economic burden. One study discovered that ageism costs the health care system in the United States approximately US $63 billion every year [23].

### Existing Research and Gaps

Thus far, there have not been any studies exploring depictions of old age in nursery rhymes. Instead, related literature has concentrated largely on portrayals of older adults in books or movies targeted at children. The most foundational of these studies is by Ansello [24], whose content analysis of more than 600 children’s books revealed that over 80% of them did not feature older characters. When older characters did appear, they were often typecast as sick, feeble, old-fashioned, out-of-touch, and unproductive. Younger characters were described with terms related to physical appearance or personality, while older characters were commonly reduced to their age. When additional descriptors were used, they usually had negative connotations, such as “poor” or “sad.” Younger characters were also portrayed as multifaceted and integral to the plot, while older characters were presented as one-dimensional and peripheral to the storyline.

Findings from Ansello’s [24] seminal study have since been corroborated by other scholars. In Hurst’s analysis of children’s picture books [25], 48% of older characters were explicitly called “old,” with common descriptors including “nice,” “funny,” “small,” “grumpy,” “lonely,” and “weak.” Dodson and Hause [26] noted that older individuals were frequently depicted as “unhealthy,” “ugly,” “eccentric,” and “passive” in books targeted at readers from kindergarten through adulthood. Janelli [27] argued that the diversity of the older demographic was not captured in picture books. In her review of over 73 storybooks, she noticed that older characters were commonly portrayed as bespectacled, having gray hair, and using walking sticks. Danowki and Robinson’s [9] analysis of more than 700 literary works published between 2000 and 2010 in the United States observed that older adults were underrepresented, though there was a marked improvement in the overall portrayal of older characters in picture books over time. Robinson et al [28], conceded that commendable effort had been made to feature more older characters in Disney films. Nonetheless, these characters remained underrepresented and mainly inhabited roles of minimal significance to the plot [28].

### This Study

From a conceptual standpoint, this study is the first to look at the ways in which old age is portrayed in nursery rhymes. Stereotypes and attitudes take hold early in life to become stable and influential guiding forces [29]. Besides affecting attitudes toward older people, age stereotypes exact a toll on one’s self-concept [3]. From a practical standpoint, this study provides insights that can inform interventions to counteract negative portrayals of old age in nursery rhymes, thus paving the way for a more age-friendly society.

Our content analysis of nursery rhymes is grounded in three research questions: To what extent is old age represented in nursery rhymes? What are the prevailing stereotypes associated with old age in nursery rhymes? Are these stereotypes primarily positive or negative?

## Methods

### Dataset

**Figure 1.**
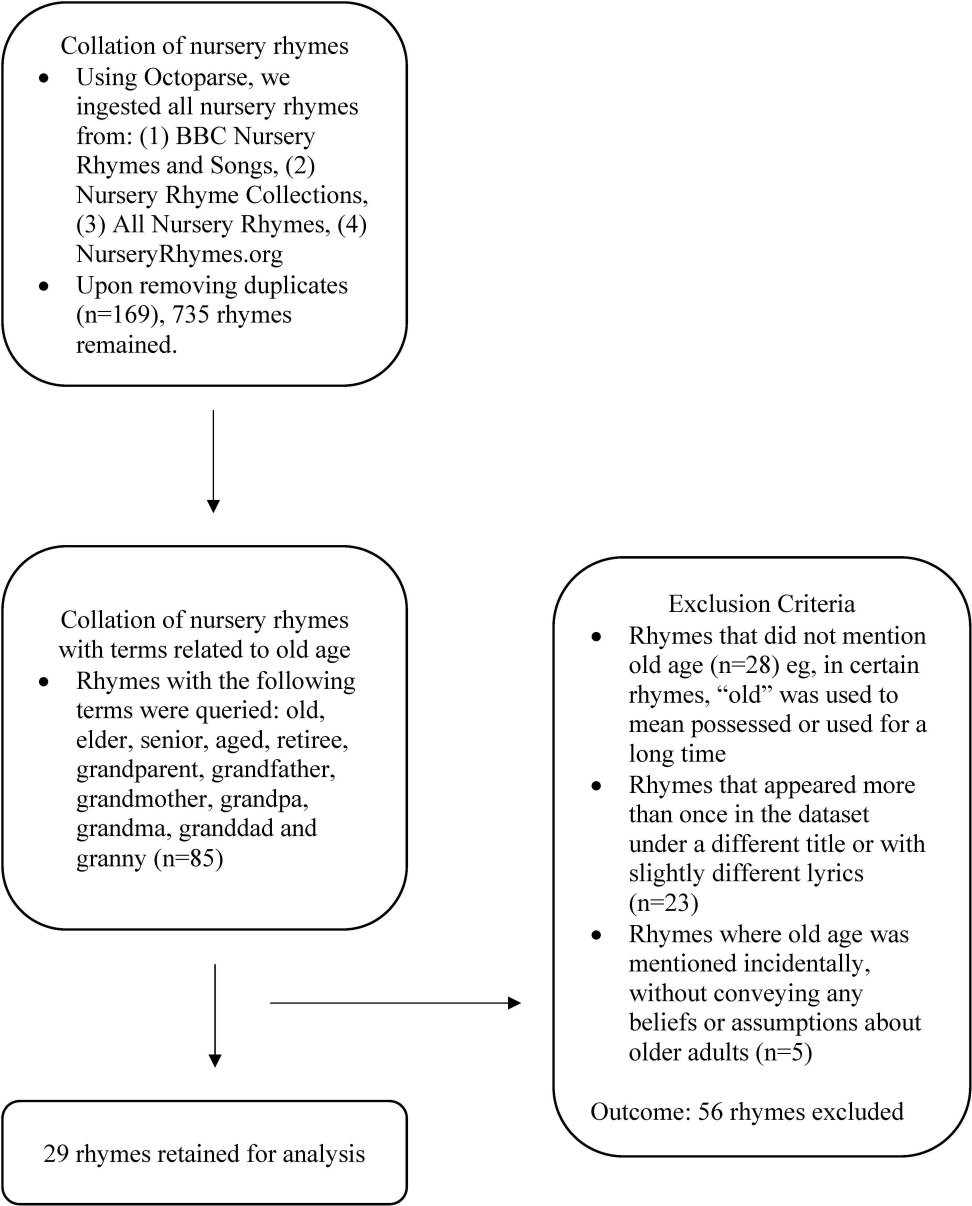
Process of collating nursery rhymes where old age was a central theme.

We gathered material from the following websites, each of which houses an extensive collection of rhymes: BBC Nursery Rhymes and Songs [30], the Nursery Rhyme Collections [31], All Nursery Rhymes [32], and NurseryRhymes.org [33]. Octoparse (Octopus Data Inc), a web-ingestion tool, was used to compile the dataset. In total, 904 nursery rhymes were retrieved. After filtering out duplicates (n=169), 735 rhymes remained. Next, to identify rhymes related to old age, we conducted a search using the following terms: old, elder, senior, aged, retiree, grandparent, grandfather, grandmother, grandpa, grandma, granddad, and granny. This search yielded a total of 85 nursery rhymes. We subsequently removed rhymes where (1) the word “old” was used to mean possessed or used for a long time rather than having lived for a long time (n=28), (2) the rhyme appeared more than once in the dataset under a different title or with minor variations in lyrics (n=23), and (3) old age was mentioned incidentally, without conveying any beliefs or assumptions about the traits of older adults, whether positive, negative, or neutral (n=5). Any rhyme that referenced “old” in relation to old age was retained even if the term was used to describe animals instead of people. This left us with 29 nursery rhymes. Figure 1 depicts the process of collecting the data.

### Content Coding of Nursery Rhymes

Following past scholarship [34-37], we conducted a thematic analysis. The coding rubric was designed using both deductive and inductive modes of reasoning [38]. We started by identifying a set of categories based on previous research [39-42] to develop a preliminary codebook. The analysis was then conducted in several stages, with the words of each rhyme read twice by the authors to ensure familiarity with the data. The aim of the first reading was to ascertain the validity of the initial set of categories, as well as to modify the codebook until all variables were refined and defined clearly. During this first reading, a new category was added whenever a rhyme featured a particular attribute that could not be suitably coded into any of the existing categories and appeared frequently enough to warrant its own category. The aim of the second reading was to make sure we had a framework sufficiently representative of the different types of rhymes so as to finalize the coding rubric. To ensure rigor in the analysis, the 2 coders had regular discussions during which any discrepancies were reviewed and adjudicated. Areas of major overlap were identified and sectioned into broader themes. The percentage agreement between the 2 raters was 93.5% and the weighted Cohen κ was 0.91 (*P*<.001), indicating high interrater reliability.

## Results

### Summary of Insights From Content Analysis

Old age was a central theme in 4% (N=29) of the 735 nursery rhymes. Of the 29 rhymes, more than half contained negative age stereotypes (17/29, 59%) and a third contained positive ones (10/29, 34%). A small proportion portrayed old age in a neutral manner (2/29, 7%; Table 1).

**Table 1.** Themes and subthemes in portrayals of old age in nursery rhymes. Percentages may not sum to exactly 100 due to rounding.

Category and themes	Percentage
Negative portrayals of old age	59
Physically debilitated	14
Unlikable	14
Helpless	10
Cognitively impaired	7
Irresponsible	7
Unhygienic	3
Incompetent	3
Positive portrayals of old age	34
Wise	17
Affectionate	10
Jovial	7
Neutral portrayals of old age	7
Aging as natural	7

### Negative Portrayals of Old Age (17/29, 59%)

Negative age stereotypes covered include being physically debilitated, cognitively impaired, unhygienic, helpless, unlikable, irresponsible, and incompetent.

#### Physically Debilitated

“Boom, Boom, Ain’t It Great to Be Crazy?” features a character named Peck, an older doctor who “(falls) down the well and (breaks) his neck.” The older man in “It’s Raining, It’s Pouring” is unable to “get up in the morning” after he “bump(s) his head.” “Michael Finnegan” revolves around a series of misadventures befalling an accident-prone older man. He “(catches) a fish and (drops) it in,” “(climbs) a tree and (barks) his shin,” and “(slides) back down and (scrapes) his skin.”

#### Cognitively Impaired

“I Know an Old Lady who Swallowed a Fly” depicts the titular character ingesting various creatures, including a “fly,” “spider,” “bird,” and “cat.” The repetitive and nonsensical nature of her actions suggests a possible decline in her mental faculties. In “Old Mother Hubbard,” old age is insinuated as being a time of forgetfulness. Old Mother Hubbard goes to the “fishmonger’s,” “grocer’s,” and “baker’s” but seemingly forgets to buy her dog food.

#### Unhygienic

Old age is equated with unkemptness in “Desperate Dan.” The eponymous character is classed as a “scruffy old man.” The portrayal of him “wash(ing) his face in a frying pan” and “comb(ing) his hair with a leg of a chair” adds to the depiction of someone who neglects personal hygiene. This eccentric behavior also suggests confusion and possibly cognitive decline or dementia.

#### Helpless

In “There was an Old Woman (As I’ve Heard Tell),” old age is positioned as a time of helplessness. After the older lady “(falls) asleep on the King’s highway” on her journey to the market, a peddler takes advantage of her by “(cutting) her petticoat up,” leaving her to “shiver and freeze.” She later wakes up disoriented and struggles to recognize herself. She says, “If it be I as I hope it may be,” her dog would be able to recognize her. When the dog barks at her, a sign it does not recognize her, she cries, “Oh dearie me.” In ”Christmas is Coming,” the line “put a penny in the old man’s hat” illustrates an impoverished older person dependent on the goodwill of others.

#### Unlikable

“The Old Woman Who Lived in a Shoe” describes a mother who provides her children “broth without any bread.” The phrase “[whipping] them all soundly” emphasizes her callous approach to parenting. “Goosey Goosey Gander” presents the older man as uncooperative as he refuses to “say his prayers,” leading the protagonist to take the former “by the left leg and [throw] him down the stairs.”

#### Irresponsible

“Old Mother Leary” portrays the protagonist as behaving impetuously. She “[leaves] a lantern in the shed,” which results in a fire when a “cow [kicks] it over.” Her wink as the fire breaks out implies she took pleasure in the ensuing chaos.

#### Incompetent

In the sea shanty “Blow the Man Down,” the “old skipper” is derided as incompetent. He is described as someone who “once played a remarkable game” but is now unable to navigate his ship, which is “becalmed in the tropical sea.” The lyrics further underscore his nautical ineptitude by stating that he “whistled all day, but in vain for a breeze,” hinting at his inability to get the ship moving.

### Positive Portrayals of Old Age (10/29, 34%)

Positive age stereotypes covered include being wise, affectionate, and jovial.

#### Wise

In “A Wise Old Owl,” the phrase “the more he saw the less he spoke, the less he spoke the more he heard” conveys that with age comes wisdom and the ability to listen and observe. The concluding line, “why can’t we all be like that wise old bird?” demonstrates that the owl’s qualities of being observant and thoughtful are worth emulating. Likewise, in “Dance Ter Yer Daddy,” old age is celebrated as a stage of life where one bequeaths knowledge and experiences to the next generation. This is evident from the lines “when yer an old man, father to a son, sing to him the old songs, sing of all you’ve done, pass along the old ways, then let his song begin, dance to your daddy, ma bonnie lamb.”

“Manx Lullaby” exalts old age as a time where the accrual of experience and knowledge offers guidance and insight. The lines “Through your life there are many, many, many tales, gems and stories you’ll be told. Remember these songs and these rhymes as you grow old; I mean, the truth will never leave you” communicate the idea that the stories and experiences one gathers throughout life should be cherished. “If All the World was Apple Pie” opens with a hypothetical inquiry, “If all the world was apple pie, and all the sea was ink, and all the trees were bread and cheese, what should we have for drink?” The subsequent line, “it’s enough to have an old man scratch his head and think,” references the intellectual prowess of older individuals by suggesting that such a perplexing question would even stump someone of advanced age.

#### Affectionate

When a caterpillar crawls on the protagonist’s grandmother in “Little Arabella Miller,” she proclaims, “Arabella Miller, how I love your caterpillar!” In “Over the River and Through the Woods,” the lines “when grandmother sees us come, she will say, “Oh, dear, the children are here, bring a pie for everyone” show a grandmother ever ready to indulge her grandchildren with treats.

#### Jovial

The central figure in “Old King Cole” is characterized as a “merry old soul.” The verse notes that he “called for his pipe and he called for his bowl and he called for his fiddlers three,” highlighting his zest for life. In “This Old Man,” the refrain “played knick-knack” captures the character’s spirited involvement in the world around him as he enthusiastically taps on different objects while engaging with others in a light-hearted manner.

### Neutral Portrayals of Old Age (2/29, 7%)

Neutral portrayals depicted old age as a natural part of life. In “Grandfather’s Clock,” the clock symbolizes the passage of the life of the protagonist’s grandfather. Significant life events are recounted, such as “when he entered at the door with a blooming and beautiful bride.” The narrative concludes without judgment, stating that the clock “stopped short, never to go again, when the old man died.” In “Do Do, Baby Do,” the line “daddy old hen dozes over ‘neath the roses, tiny chicks she’ll have for you” situates old age within the larger context of family life. It suggests that aging is part of a natural process, with one generation giving way to the next.

## Discussion

### Principal Findings

This study delved into representations of old age in nursery rhymes. We found that only 4% of the nursery rhymes compiled mentioned old age. Of these, over half presented old age negatively, while about a third presented it positively. These findings comport with past literature about age stereotypes being multidimensional, albeit with a tendency toward negativity [14-16,43].

Considering the widespread tendency to overlook the experiences and contributions of older adults, it is unsurprising—though no less concerning—that just 4% of the nursery rhymes mentioned old age. This figure is especially stark given that by 2030, one in 6 people globally will be at least 60 years of age [44]. The issue of underrepresenting or, worse, excluding the older demographic has been flagged in other forms of children’s media [9,24-28] as well as popular culture in general [45-48]. This erasure of older adults in the media in turn contributes to their invisibility in wider society.

The presence of negative age stereotypes in more than half of the rhymes raises important concerns about their potential impact on children’s beliefs. It has been established that children tend to harbor certain negative stereotypes of older adults [39,40,42]. As per the theory of cultivation, repeated exposure to negative messages about older adults can influence children’s perceptions of reality [7-11]. Besides perpetuating ageism and stymying the development of intergenerational solidarity, it also impacts them personally. From the perspective of the communicative ecology model of successful aging [12], negative portrayals of older adults can affect children’s self-efficacy regarding their aging process. In addition, consistent with the theory of stereotype embodiment, these narratives can have detrimental effects on their mental and physical health [22].

Old age was cast in a positive light in 34% of the rhymes. These positive depictions offer a much-needed counterbalance to the ageist portrayals replete in popular culture [36,49-52]. In the endeavor to enhance representations of old age, it is crucial to bear in mind that the objective should not be for all rhymes to exclusively endorse positive age stereotypes. Overly romanticized portrayals of old age may wind up creating unrealistic expectations not in sync with the diverse realities of the aging experience [53-56]. Instead, the goal should be to portray old age in a balanced and authentic manner that acknowledges the heterogeneity of the older cohort, as well as the complexities inherent in the aging process [57].

### Practical Implications

We propose a set of interventions to improve portrayals of old age in nursery rhymes.

First, new nursery rhymes could be composed to challenge negative age stereotypes. Jan Golden’s creation of “Age-Friendly Vibes,” a line of greeting cards developed to counter the ageism rampant in birthday cards, is an example of how new forms of expression are necessary to disrupt ageist thinking [58,59]. The creation of new rhymes could involve collaborations among writers, educators, creative professionals, gerontologists, and older adults themselves. These rhymes could be incorporated into educational materials, children’s books, and digital platforms to ensure widespread accessibility and exposure. Efforts could also be made to revise existing nursery rhymes to depict the older population more positively, though it is essential to consider how such modifications might be received by educators and parents.

Second, curriculum developers should be mindful not to perpetuate negative attitudes toward older adults. There is evidence that stereotypes often take hold in one’s childhood years and carry on into adulthood [29]. Educational materials should therefore be thoughtfully designed to foster understanding toward older individuals.

Third, intergenerational storytelling and performance sessions could be organized in schools, libraries, community centers, or senior living facilities. These sessions would create opportunities for older individuals to share their stories, experiences, and talents with their younger contemporaries. Crucially, these sessions encourage the reciprocal exchange of knowledge and skills while also enhancing the well-being of both groups [60,61].

Fourth, introducing opportunities for collaborative projects across age groups could bridge generational gaps. By encouraging joint artistic endeavors, both generations can contribute their perspectives, talents, and insights, thereby facilitating intergenerational learning [60,61].

Finally, there is a need to provide parents and caregivers with educational resources on ageism. As these individuals usually select the materials children are exposed to, it is crucial that they choose content that advances a more inclusive view of aging. Equipping them with the resources to challenge ageism will create a lasting impact on children’s attitudes toward aging.

### Limitations and Future Directions

This study has several limitations. First, since most of the rhymes in our dataset were written decades or even centuries ago, the sentiments expressed may be more representative of past attitudes than contemporary ones. Nonetheless, these rhymes warrant scrutiny for their continued relevance today. In addition, we did not manage to locate the country of origin and year of publication for all the rhymes, which limits our ability to fully contextualize them. Second, most, if not all, of the nursery rhymes in our dataset seem to have originated in the West, which means the depictions of old age likely reflect Western perspectives. Since norms of old age vary across cultures [62,63], future studies could investigate the depictions of old age in popular children’s poems or songs from other countries. Third, as we were unable to determine authorial intent, our interpretation of each rhyme was inherently subjective. Nonetheless, the high interrater reliability supports the robustness of our conclusions.

To build on the findings of this study, subsequent investigations could dive into the illustrations that accompany nursery rhymes in books or videos. This will generate insights into the visual cues and symbols associated with old age in nursery rhymes. Exploring children’s perceptions of depictions of older adults in nursery rhymes is another line of research worth pursuing. It would also be valuable to conduct a historical analysis of rhymes to determine whether newer rhymes contain more negative stereotypes than older ones, for instance. Finally, future research could compare nursery rhymes or other content targeted at children across different societies to examine whether there are cultural differences in the way aging is portrayed.

### Conclusion

In the context of an aging population, it is paramount that people embrace a less pessimistic outlook on aging. Although nursery rhymes may seem like mere tales not to be taken seriously, they are powerful cultural artifacts capable of molding thought processes. Our study highlights the need to give children access to more accurate and nuanced stories about older adults. By doing so, society can move beyond passively assimilating negative views of aging to actively fostering healthier ones, thus building a future where all are valued regardless of age.

## References

[1] Officer A, Thiyagarajan JA, Schneiders ML, Nash P, de la Fuente-Núñez V (2020). Ageism, healthy life expectancy and population ageing: how are they related?. Int J Environ Res Public Health.

[2] Butler RN (1969). Age-ism: another form of bigotry. Gerontologist.

[3] Levy B (2009). Stereotype embodiment: a psychosocial approach to aging. Curr Dir Psychol Sci.

[4] Bellingtier JA, Schenker LE, Weber AL (2024). Judging a book by its older adult cover: age-related expectations and parental preference for children’s books. Curr Psychol.

[5] Patton DA (2021). We all fall down: head injuries in nursery rhyme characters. BMJ.

[6] Mullen G (2017). More than words: using nursery rhymes and songs to support domains of child development. J Child Stud.

[7] Gerbner G, Gross L, Morgan M, Signorielli N, Shanahan J, Bryant J, Zillmann D (2002). Media Effects: Advances in Theory and Research.

[8] Gerbner G, Morgan M, Signorielli N, Bryant J, Zillman D (1986). Perspectives on Media Effects Hilldale.

[9] Danowski J, Robinson T (2012). The portrayal of older characters in popular children’s picture books in the US. J Child Media.

[10] Shrum J, Bischak D (2001). Mainstreaming, resonance, and impersonal impact. Human Comm Res.

[11] Gerbner G, Gross L, Signorielli N, Morgan M (1980). Aging with television: images on television drama and conceptions of social reality. J Commun.

[12] Fowler C, Gasiorek J, Giles H (2015). The role of communication in aging well: introducing the communicative ecology model of successful aging. Commun Monogr.

[13] Gettings PE, Kuang K (2022). Extending the communicative ecology model of successful aging using talk about careers and retirement. Commun Monogr.

[14] Aday RH, Aday KL, Arnold JL, Bendix SL (1996). Changing children’s perceptions of the elderly: the effects of intergenerational contact. Gerontol Geriatr Educ.

[15] Rich PE, Myrick RD, Campbell C (1983). Changing children’s perceptions of the elderly. Educ Gerontol.

[16] McTavish DG (1971). Perceptions of old people: a review of research methodologies and findings. Gerontologist.

[17] Isaacs LW, Bearison DJ (1986). The development of children’s prejudice against the aged. Int J Aging Hum Dev.

[18] Levy BR, Hausdorff JM, Hencke R, Wei JY (2000). Reducing cardiovascular stress with positive self-stereotypes of aging. J Gerontol B Psychol Sci Soc Sci.

[19] Levy BR, Slade MD, Kasl SV (2002). Longitudinal benefit of positive self-perceptions of aging on functional health. J Gerontol B Psychol Sci Soc Sci.

[20] Brothers A, Kornadt AE, Nehrkorn-Bailey A, Wahl HW, Diehl M (2021). The effects of age stereotypes on physical and mental health are mediated by self-perceptions of aging. J Gerontol B Psychol Sci Soc Sci.

[21] Chang ES, Kannoth S, Levy S, Wang SY, Lee JE, Levy BR (2020). Global reach of ageism on older persons’ health: a systematic review. PLOS ONE.

[22] Levy B (2009). Stereotype embodiment. Curr Dir Psychol Sci.

[23] Levy BR, Slade MD, Chang ES, Kannoth S, Wang SY (2020). Ageism amplifies cost and prevalence of health conditions. Gerontologist.

[24] Ansello EF (1977). Age and ageism in children’s first literature. Educ Gerontol.

[25] Hurst J (1981). Images in Children’s Picture Books.

[26] Dodson AE, Hause JB (1981). Teaching and Learning about Aging Project, McCarthy-Towne School, Charter Road, Acton, MA 01720.

[27] Janelli LM (1988). Depictions of grandparents in children’s literature. Educ Gerontol.

[28] Robinson T, Callister M, Magoffin D, Moore J (2007). The portrayal of older characters in Disney animated films. J Aging Stud.

[29] Klausmeier HJ, Goodwin W (1966). Learning and Human Abilities: Educational Psychology.

[30] BBC nursery rhymes and songs - a to z BBC school radio. BBC School Radio.

[31] All nursery rhymes in alphabetical order. The Nursery Rhyme Collections.

[32] Nursery rhymes – popular nursery rhymes with lyrics. All Nursery Rhymes.

[33] NurseryRhymes.org - nursery rhymes with lyrics and music. NurseryRhymes.org.

[34] Ng R, Indran N (2022). Not too old for TikTok: how older adults are reframing aging. Gerontologist.

[35] Ng R, Indran N (2024). Questions about aging and later life on Quora. Gerontologist.

[36] Ng R, Indran N (2023). Does age matter? Tweets about gerontocracy in the United States. J Gerontol.

[37] Ng R, Indran N (2025). Public interest in research on aging: analysis of altmetric attention scores over 5 years. J Appl Gerontol.

[38] Armat MR, Assarroudi A, Rad M, Sharifi H, Heydari A Inductive and deductive: ambiguous labels in qualitative content analysis. TQR.

[39] Goldman RJ, Goldman JDG (1981). How children view old people and ageing: a developmental study of children in four countries. Aust J Psychol.

[40] Lynott PP, Merola PR (2007). Improving the attitudes of 4th graders toward older people through a multidimensional intergenerational program. Educ Gerontol.

[41] Seefeldt C, Jantz RK, Galper A, Serock K (1977). Using pictures to explore children’s attitudes toward the elderly. Gerontologist.

[42] Weinberger A (1979). Stereotyping of the elderly: elementary school children’s responses. Res Aging.

[43] Ng R, Indran N, Liu L (2024). Social media discourse on ageism, sexism, and racism: analysis of 150 million tweets over 15 years. J Am Geriatr Soc.

[44] (2021). Ageing and health. World Health Organization.

[45] Bishop JM, Krause DR (1984). Depictions of aging and old age on Saturday morning television. Gerontologist.

[46] Laughlin CA (2019). Images of Aging and the Aesthetic of Actuality in Chinese Film: Reportage, Documentary, and the Art of the Real. Mod Chin Lit Cult.

[47] Lien SC, Zhang YB, Hummert ML (2009). Older adults in prime-time television dramas in Taiwan: prevalence, portrayal, and communication interaction. J Cross Cult Gerontol.

[48] Shary T, McVittie N (2016). Fade to Gray: Aging in American Cinema.

[49] Berger R (2017). Aging in America: ageism and general attitudes toward growing old and the elderly. JSS.

[50] Indran N, Ng R (2025). Artistic provocation or ageist stereotyping?. J Am Geriatr Soc.

[51] Ng R, Indran N (2024). Age advocacy on Twitter over 12 years. Gerontologist.

[52] Ng R, Indran N (2023). Videos about older adults on TikTok. PLOS ONE.

[53] Ng R, Indran N (2024). Reframing aging: foregrounding familial and occupational roles of older adults is linked to decreased ageism over two centuries. J Aging Soc Policy.

[54] Ng R, Indran N (2023). Impact of old age on an occupation’s image over 210 years: an age premium for doctors, lawyers, and soldiers. J Appl Gerontol.

[55] Ng R, Indran N (2024). Youth is prized in medicine, old age is valued in law: analysis of media narratives over 200 years. J Med Internet Res.

[56] Ng R, Indran N, Liu L (2024). Advocating for older adults in the age of social media: strategies to achieve peak engagement on Twitter. JMIR Aging.

[57] Ng R, Indran N (2022). Role-based framing of older adults linked to decreased ageism over 210 years: evidence from a 600-million-word historical corpus. Gerontologist.

[58] Williams M (2024). Denver designer unveils range of birthday cards with positive messages about getting older after claiming “damn, you’re old” type rivals are driving the elderly into an early grave. Daily Mail Online.

[59] Lin SSH, Walden A (2024). Ageism in birthday cards: a mixed-method content analysis. Gerontologist.

[60] Boger J, Mercer K (2017). Technology for fostering intergenerational connectivity: scoping review protocol. Syst Rev.

[61] Burnes D, Sheppard C, Henderson CR (2019). Interventions to reduce ageism against older adults: a systematic review and meta-analysis. Am J Public Health.

[62] Ng R, Indran N (2021). Societal perceptions of caregivers linked to culture across 20 countries: evidence from a 10-billion-word database. PLOS ONE.

[63] Ng R, Indran N (2024). #ProtectOurElders: analysis of Tweets about older Asian Americans and anti-Asian sentiments during the COVID-19 pandemic. J Med Internet Res.

